# Publisher Correction: Histones with an unconventional DNA-binding mode in vitro are major chromatin constituents in the bacterium *Bdellovibrio bacteriovorus*

**DOI:** 10.1038/s41564-024-01730-w

**Published:** 2024-05-22

**Authors:** Antoine Hocher, Shawn P. Laursen, Paul Radford, Jess Tyson, Carey Lambert, Kathryn M. Stevens, Alex Montoya, Pavel V. Shliaha, Mathieu Picardeau, R. Elizabeth Sockett, Karolin Luger, Tobias Warnecke

**Affiliations:** 1 Medical Research Council London Institute of Medical Sciences, London, UK; 2 Institute of Clinical Sciences, Faculty of Medicine, Imperial College London, London, UK; 3 Department of Molecular, Cellular, and Developmental Biology, University of Colorado Boulder, Boulder, CO USA; 4 School of Life Sciences, Medical School, Queen’s Medical Centre, University of Nottingham, Nottingham, UK; 5 Institut Pasteur, Université Paris Cité, CNRS UMR 6047, Biology of Spirochetes Unit, Paris, France; 6 Department of Biochemistry, University of Colorado Boulder, Boulder, CO USA; 7 Howard Hughes Medical Institute, Chevy Chase, MD USA

**Keywords:** Chromatin structure, Molecular evolution, X-ray crystallography

Correction to: *Nature Microbiology* 10.1038/s41564-023-01492-x, published online 9 October 2023

In the version of the article initially published, the labels in Fig. 6a, now reading “*Aquifex aeolicus*” and “*Thermus thermophilus*”, originally read “RNA (this study, aq_328 & aq_616)” and “RNA (ref.^65^, TTC1115)”. The figure has now been corrected in the HTML and PDF versions of the article.

