# Supplementary material for: Histones with an unconventional DNA-binding mode in vitro are major chromatin constituents in the bacterium Bdellovibrio bacteriovorus
Source: Nat Microbiol. 2023 Oct 9;8(11):2006–19. doi: 10.1038/s41564-023-01492-x (PMC10627809; doi:10.1038/s41564-023-01492-x)
Supplement: Supplementary file 1 — Supplementary Figs. 1–16 and Table 2. [file 41564_2023_1492_MOESM1_ESM.pdf]

nature microbiology

Article https://doi.org/10.1038/s41564-023-01492-x

Histones with an unconventional DNA-binding mode in vitro are major chromatin constituents in the bacterium *Bdellovibrio bacteriovorus*

In the format provided by the authors and unedited

Supplementary Material

for

"Histones with an unconventional DNA binding mode are major chromatin constituents in the bacterium *Bdellovibrio bacteriovorus*"

(Hocher, Laursen, *et al.* 2023)

This supplement contains Figures S1-16, Table S2, and Supplementary References

Tables S1,S3, and S4, Movies S1&S2, and Model S1 can be found as separate files in the supplementary material online.

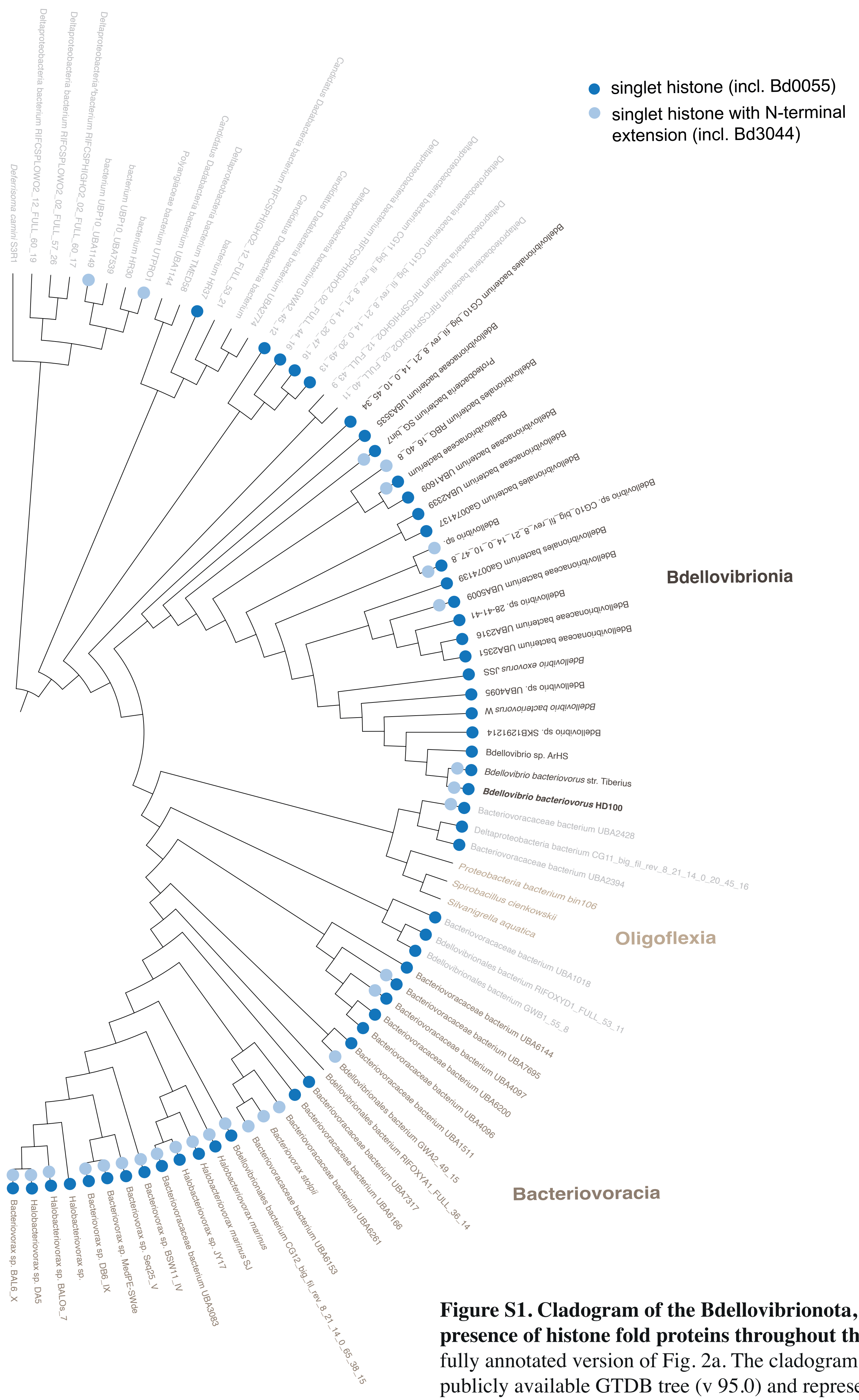

Figure S1. Cladogram of the Bdellovibrionota, indicating the presence of histone fold proteins throughout the clade. This is a fully annotated version of Fig. 2a. The cladogram is based on the publicly available GTDB tree (v 95.0) and represents the subset of genome assemblies that were used in this study and are also present in GTDB.

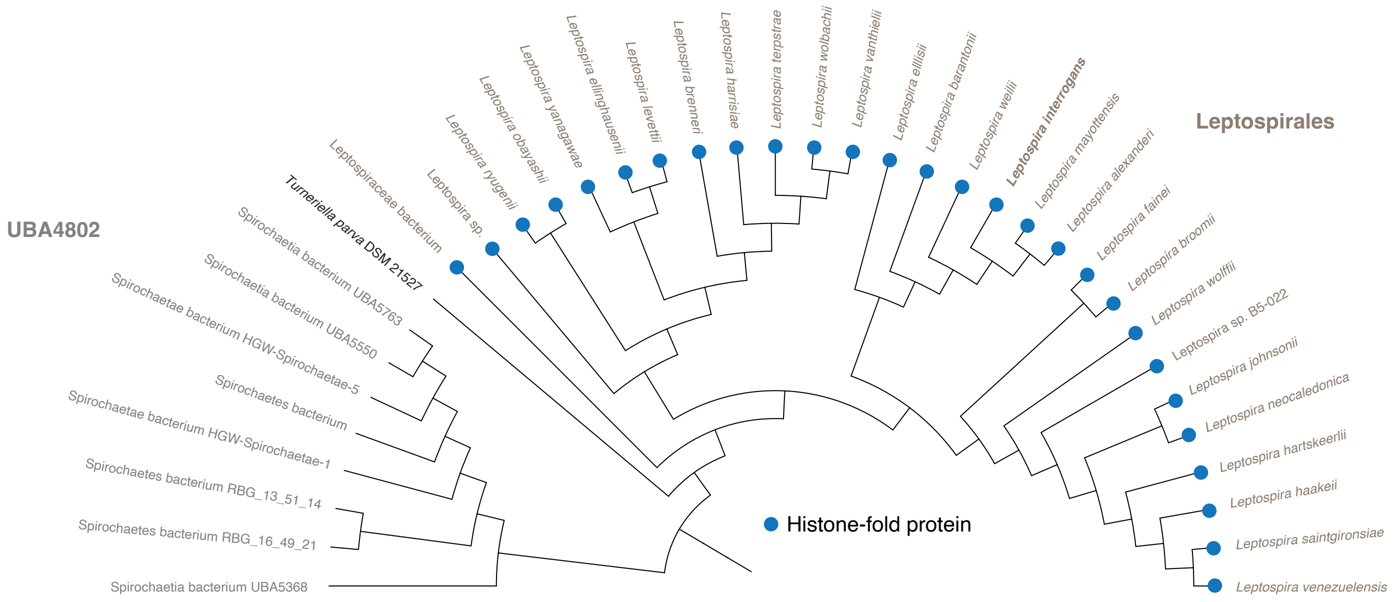

Figure S2. Cladogram of the class Leptospirales and its UBA4802 sister clade, indicating the presence of histone fold proteins throughout the clade. The cladogram is based on the publicly available GTDB tree (v 95.0) and represents the subset of genome assemblies that were used in this study and are also present in GTDB.

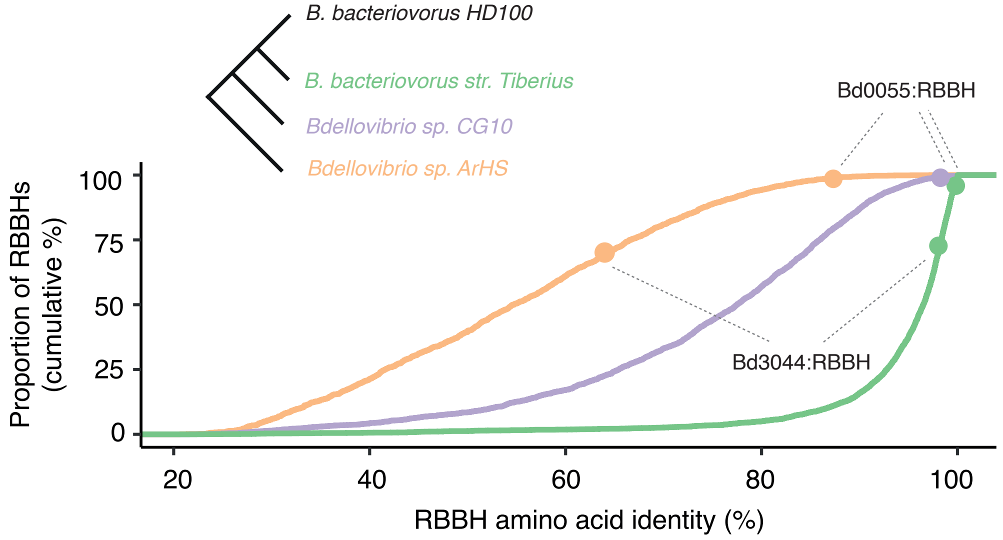

Figure S3. Conservation of Bd0055 at the amino acid level in the context of other proteins encoded by *B. bacteriovorus*. Homologs of proteins in *B. bacteriovorus* HD100 were identified in three other Bdellovibrio genomes of different divergence levels (*B. bacteriovorus* strain Tiberius, Bdellovibrio sp. CG10, and Bdellovibrio sp. ArHS) using a reciprocal best blast hit (RBBH) approach (see Methods). For each pairwise comparison, proteins were aligned and then ranked by amino acid identity. Bd0055 ranks amongst the most highly conserved proteins at different levels of proteome divergence. Bd3044 is comparatively less well conserved and absent from Bdellovibrio sp. CG10 (assembly ID: GCA_002773975.1).

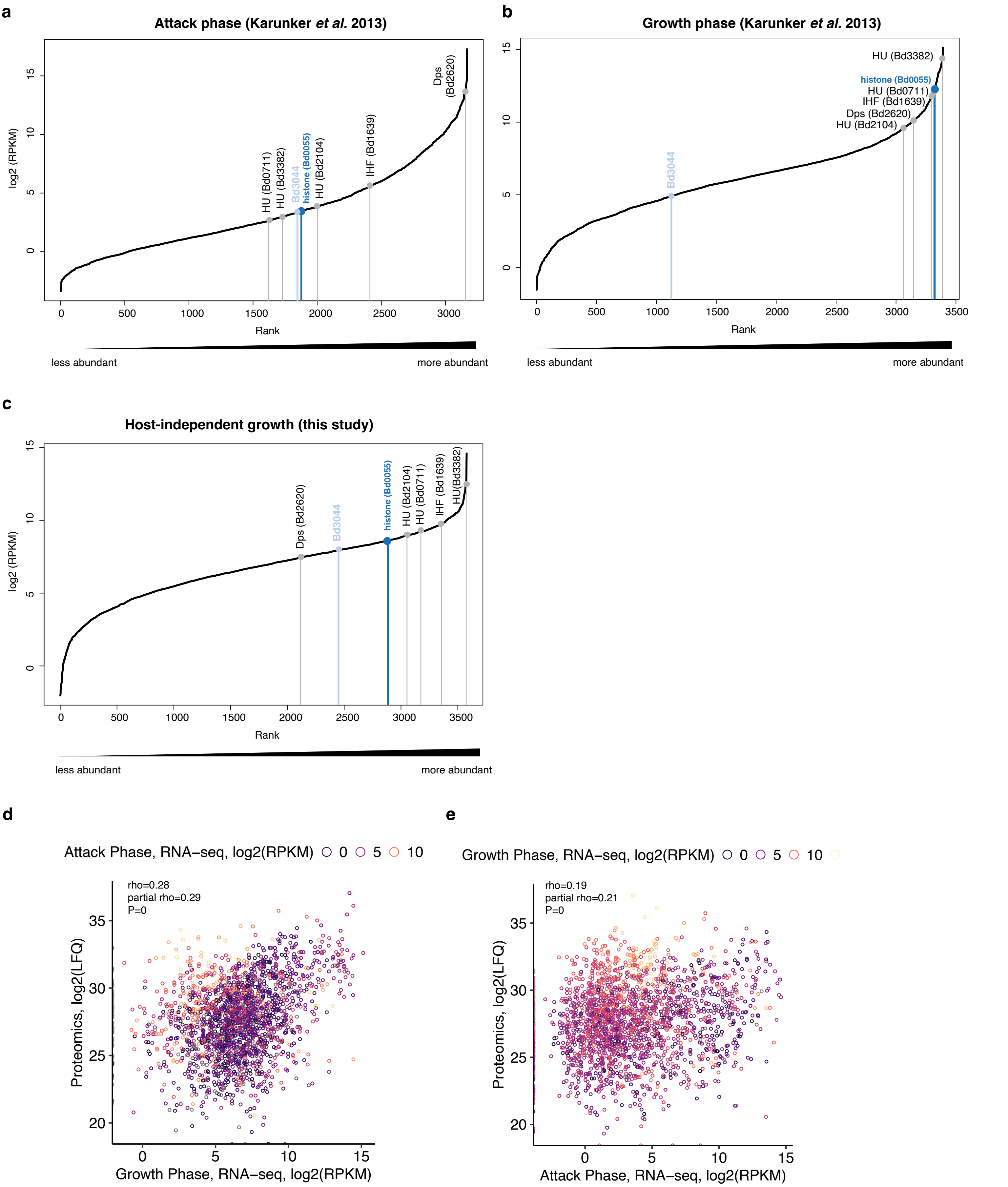

Figure S4. Ranked abundance of *B. bacteriovorus* transcripts during **(a)** attack phase, **(b)** growth inside the *E. coli* host, and **(c)** host-independent growth (strain HID13), highlighting transcript levels of Bd0055 relative to homologs of classic bacterial NAPs encoded in the *B. bacteriovorus* genome. **(d, e)** Correlation between protein abundance in attack phase cells (this study, whole cell extract) and RNA abundance during attack phase and growth inside the host (from Karunker *et al*. 2013, ref. 66).

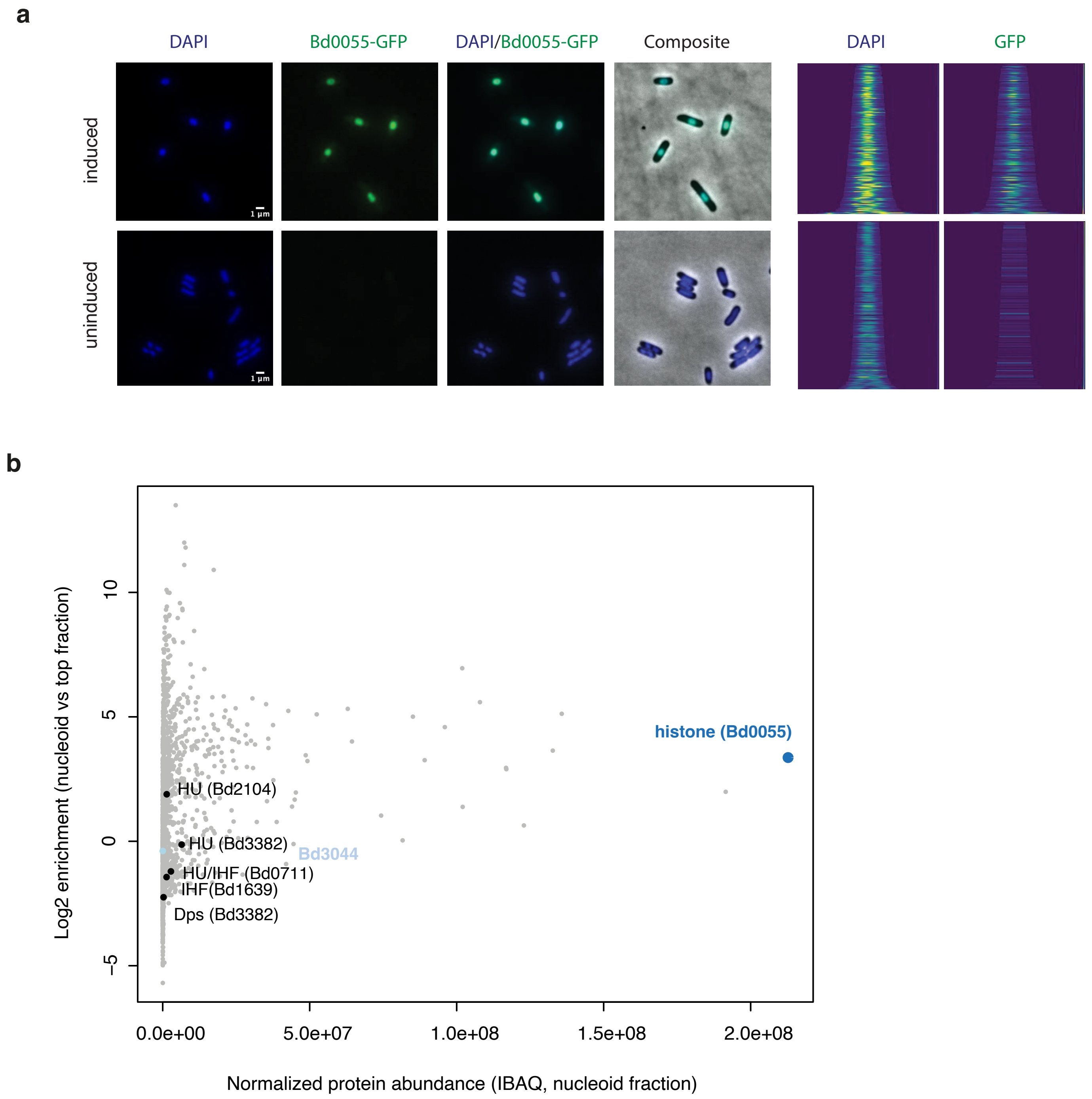

Figure S5. Bd0055 associates with the nucleoid. (**a**) A Bd0055-GFP fusion protein expressed in *E. coli* K-12 (see Methods) co-localizes with the nucleoid, which is visualized by DAPI staining. Representative images from experiments done in triplicate are shown. (**b**) Protein abundance and nucleoid enrichment of Bd0055 in *B. bacteriovorus* in the context of "classic" bacterial NAPs, for which homologs were detected in the *B. bacteriovorus* HD100 genome.

a

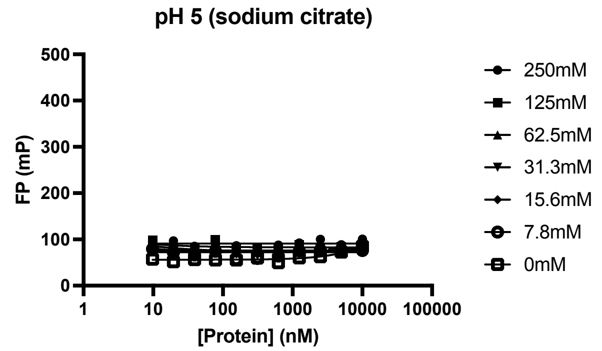

	250mM	125mM	62.5mM	31.3mM	15.6mM	7.8mM	0mM
IC50	~ 4.930e-032	2.005	4.533	5.014	Unstable	Unstable	15681
HillSlope	9.631e-010	-0.8437	-0.9899	-2.503	Unstable	Unstable	-1.220

	250mM	125mM	62.5mM	31.3mM	15.6mM	7.8mM	0mM
R squared	0.6442	0.6506	0.8779	0.7410	0.07907	-0.002774	0.2236

b

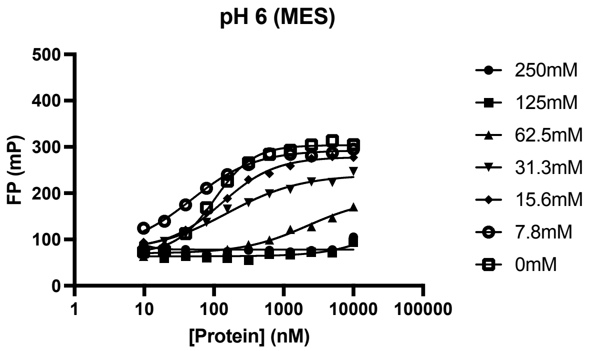

	250mM	125mM	62.5mM	31.3mM	15.6mM	7.8mM	0mM
IC50	9.850	32664	2196	154.7	130.1	47.62	102.6
HillSlope	4.018	-1.082	0.9452	0.8278	1.137	0.9560	1.563

	250mM	125mM	62.5mM	31.3mM	15.6mM	7.8mM	0mM
R squared	0.3220	0.2752	0.9434	0.9894	0.9949	0.9963	0.9951

c

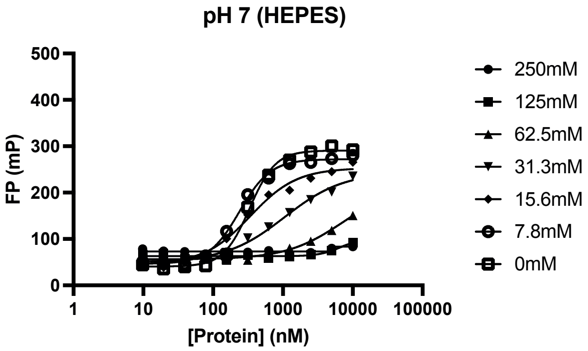

	250mM	125mM	62.5mM	31.3mM	15.6mM	7.8mM	0mM
IC50	Unstable	6480	7331	1059	341.2	238.5	332.2
HillSlope	33.57	2.846	1.062	1.032	1.307	1.950	2.338

	250mM	125mM	62.5mM	31.3mM	15.6mM	7.8mM	0mM
R squared	0.3678	0.7255	0.9731	0.9869	0.9860	0.9962	0.9948

d

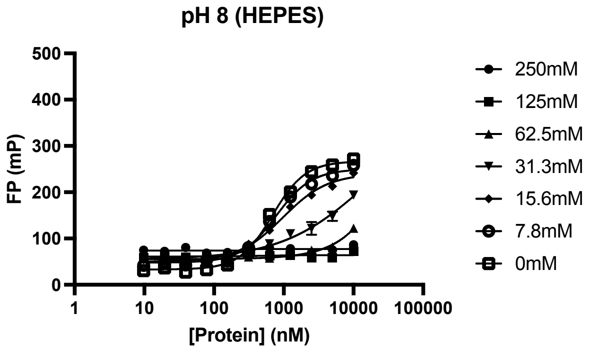

	250mM	125mM	62.5mM	31.3mM	15.6mM	7.8mM	0mM
IC50	Unstable	467.9	Unstable	15360	967.7	776.6	687.5
HillSlope	Unstable	-1.584	1.071	0.7112	1.350	1.700	1.836

	250mM	125mM	62.5mM	31.3mM	15.6mM	7.8mM	0mM
R squared	0.07528	-0.01476	0.9459	0.9865	0.9931	0.9897	0.9940

Figure S6. Fluorescence polarization (FP) was used to monitor interaction of Bd0055 with 40 bp DNA labelled with Alexa-488 labeled, at the indicated pH and NaCl concentration (a-d). Binding affinities are listed below each condition. All experiments were carried out in triplicate. Error bars represent standard error of the mean.

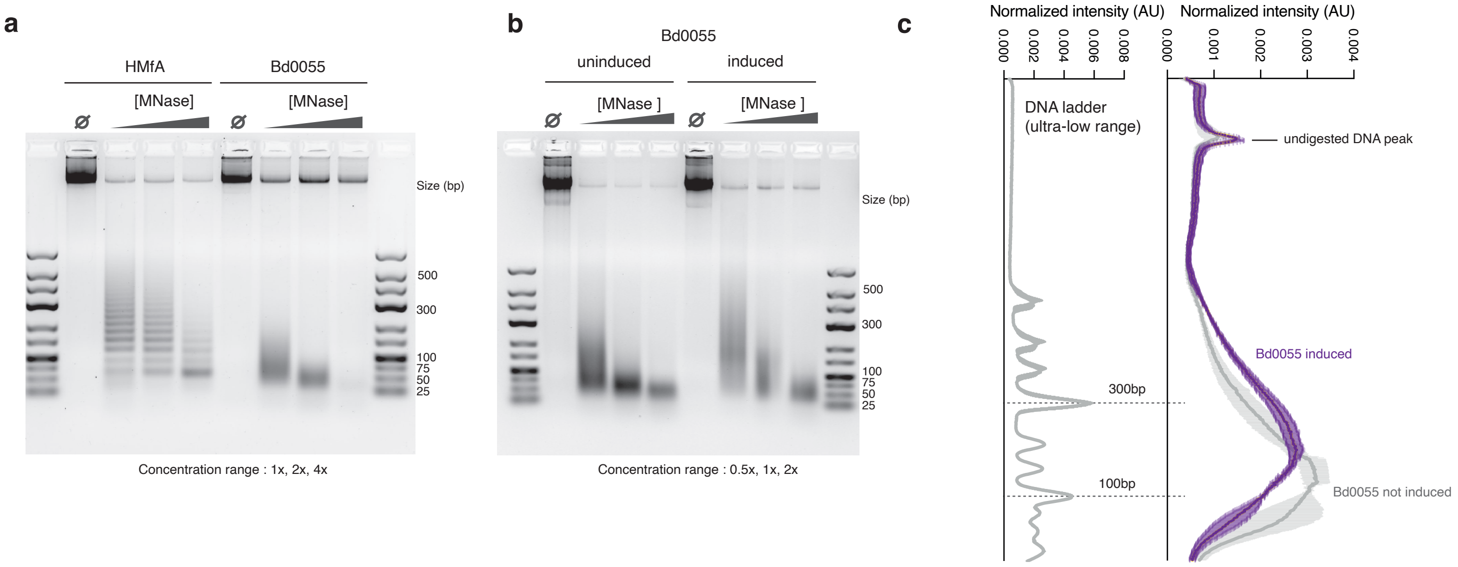

Figure S7. Micrococcal nuclease (MNase) digestion of chromatin from *E. coli* strains heterologously expressing either the archaeal histone HMfA or B0055. (**a**) In cells expressing HMfA, MNase digestion results in a ladder of protected fragments consistent with hypernucleosome formation. No such banding pattern is evident when Bd0055 is expressed instead. Note that areas of strong signal in Bd0055 lanes could be misread to imply the existence of a relatively defined fragment size class. This is not the case and rather reflects the fact that genomic DNA is quickly digested to low molecular weight species that accumulate at the bottom of the gel. This is evident from the fact that we observe a similar banding pattern when digesting chromatin from the same strain but where Bd0055 was not induced (**b**). Expression in the uninduced condition is, in fact, strongly repressed. Note also that – while fragments of a defined size are absent – there is greater protection (visible as smears centred at higher molecular weight in the induced condition) when Bd0055 is present, as quantified in (**c**), showing mean and standard error of the mean for three replicates at an MNase concentration of 0.5x. Finally, we note that these patterns allow no firm conclusions regarding a role of Bd0055 in protecting or regulating access to DNA. All we can say is that Bd0055 does not protect *from MNase* in the same manner that archaeal and eukaryotic histone complexes do. MNase probes accessibility in a specific way, and there are various proteins, including for example H-NS in *E. coli*, that prevent access to DNA, but do not confer MNase protection.

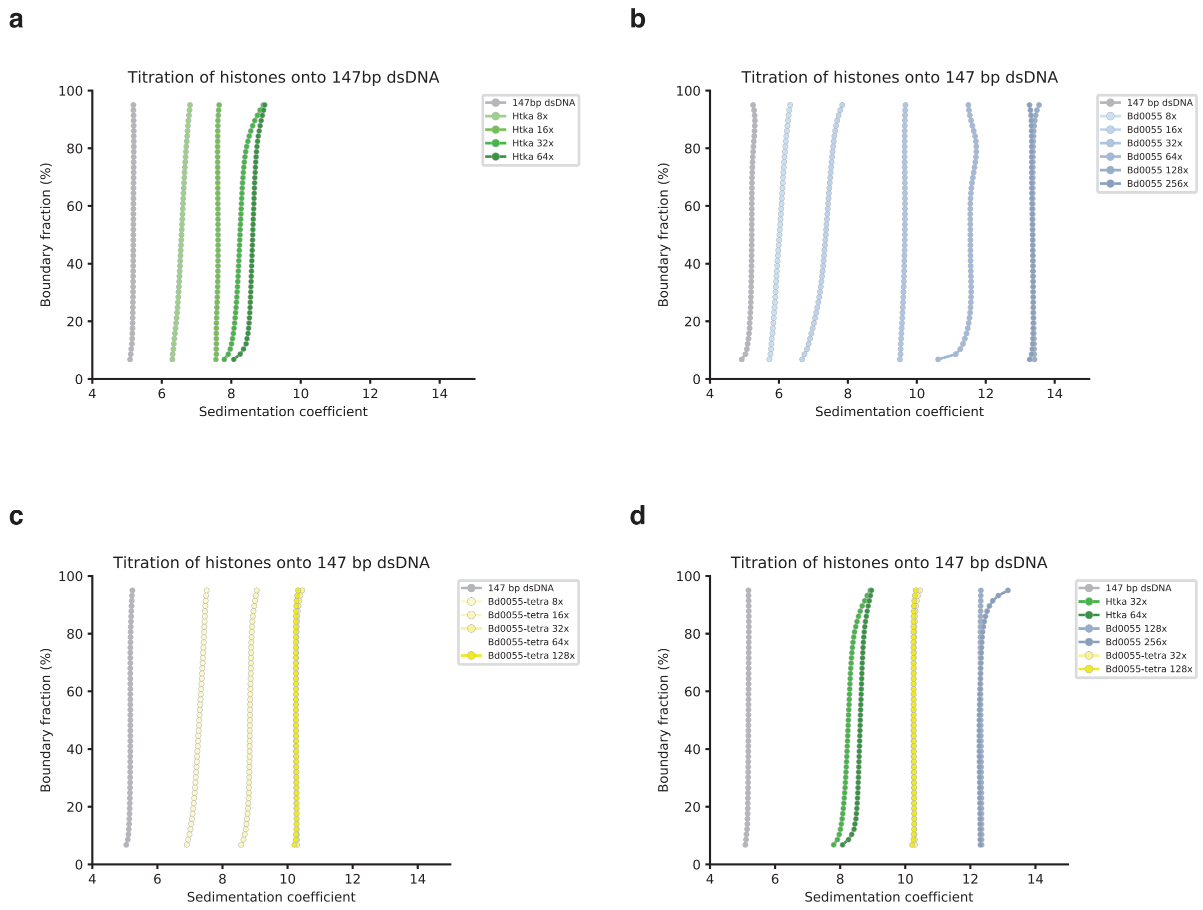

Figure S8. Sedimentation behavior of Bd0055 and HTkA DNA complexes indicates large Bd0055-DNA complexes. (**a**) SV-AUC of a titration of HTkA onto 147 bp DNA. Absorbance is represented in a van Holde-Weischet plot. HTkA saturates the piece of DNA at a molar ratio of histone monomers to DNA of ~32. Particles are insoluble beyond a 64-fold excess of histone monomer over DNA. (**b**) Titration of Bd0055 onto 147 bp DNA. Bd0055 saturates binding sites on DNA at a molar ratio of histone monomer to DNA above 128x. Particles are soluble at all concentrations measured. (**c**) Titration of Bd0055-tetra mutant onto 147 bp DNA. Bd0055-tetra saturates the piece of DNA at a molar ratio of histone monomer to DNA of <32. Particles are not soluble at 64x, but are soluble again at 128x, presumably forming a similar particle to that of the 32x sample. Particles are insoluble at 256-fold excess. (**d**) Comparison of saturating concentrations of HTkA, Bd0055, and Bd0055-tetra (select data from (a-c)).

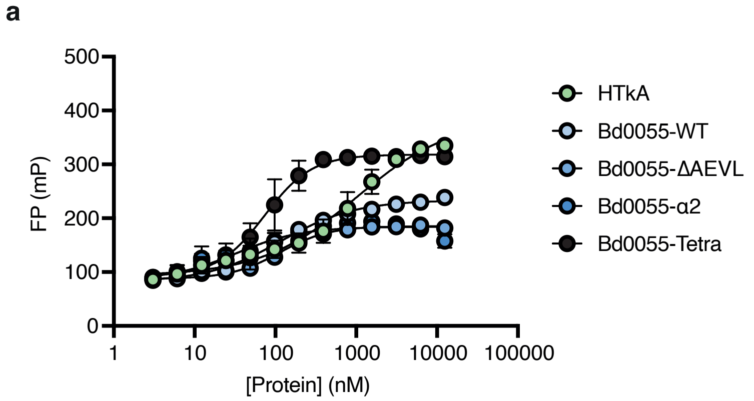

	HTkA	Bd0055-WT	Bd0055-ΔAEVL	Bd0055-α2	Bd0055-Tetra
IC50	1011	117.1	113.0	32.76	73.87
HillSlope	0.7363	0.9448	1.425	1.084	1.490

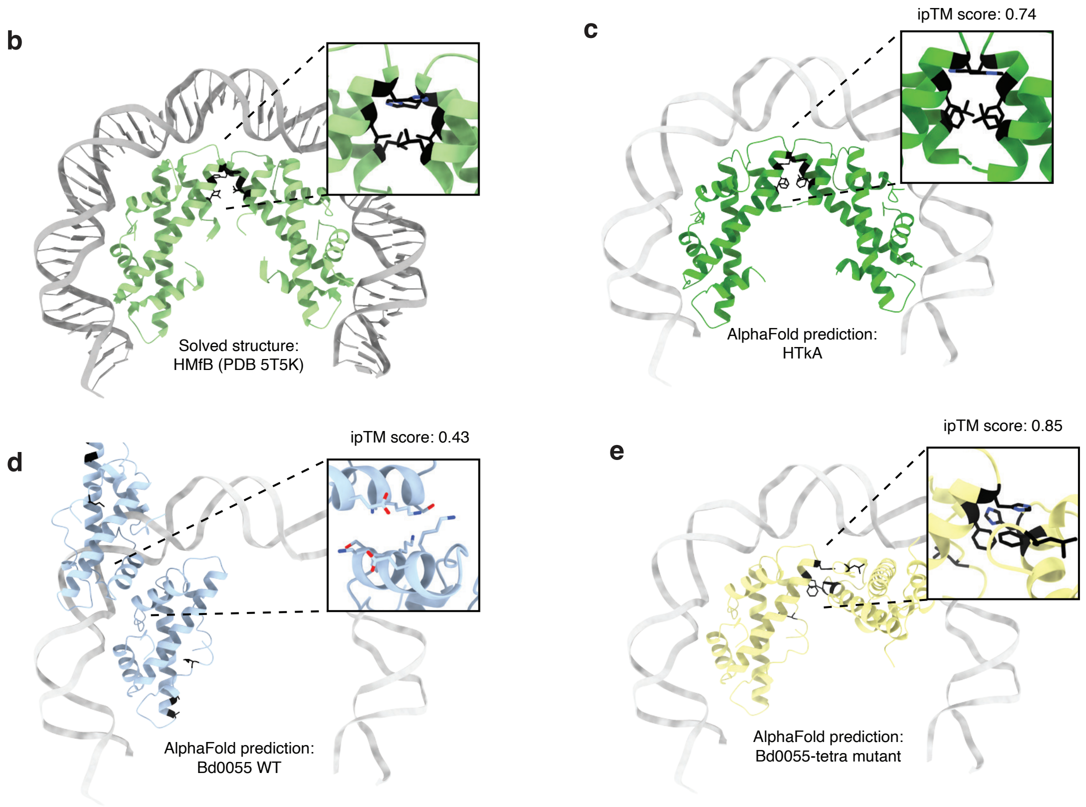

Figure S9. (a) Fluorescence polarization (FP) of Bd0055 mutants binding to the same DNA as in Fig. 5d. FP is monitored using the Alexa488 flourophore (N=3). Error bars represent standard deviation of the mean.
(b-e) Tetramerization interfaces of archaeal and bacterial histones. (**b**) Crystal structure of HMfB in complex with DNA (PDB 5T5K), only showing one tetramer of protein (green) and the DNA it interacts with (gray). The inset shows the tetramerization interface (L46, H49, L62 are highlighted in black). In addition to hydrophobic contacts by the three highlighted residues H49 may hydrogen bond with the neighboring dimer's backbone. Panels (c-e) are AlphaFold models are aligned to the same dimer of HMfB. The DNA from a) is superimposed onto each model and was not considered in AlphaFold's prediction or modeled separately. Interface predicted TM (ipTM) scores provide a measure of confidence in the interface prediction, with values above ~0.75 suggested to be high confidence (see Yin *et al*. 2022)[67]. (**c**) AlphaFold prediction of the tetramer configuration of the archaeal histone HTkA (dark green). The tetramer interface is shown enlarged in the inset (L47, H50, L63 are highlighted in black). (d) AlphaFold prediction of tetramer configuration of Bd0055 WT (blue). The predicted tetramer interface is incompatible with the DNA-protein complex architecture observed in (b,c) and unlikely to form in practice, as suggested by the low ipTM score. (e) AlphaFold prediction of tetramer configuration of the Bd0055-tetra mutant, showing tetramer interface in inset (L45, H48, L61 are highlighted in black). The tetramer is bent more severely than in (b) or (c), but its interface exhibits similarities to interfaces observed/modeled for archaeal histones.

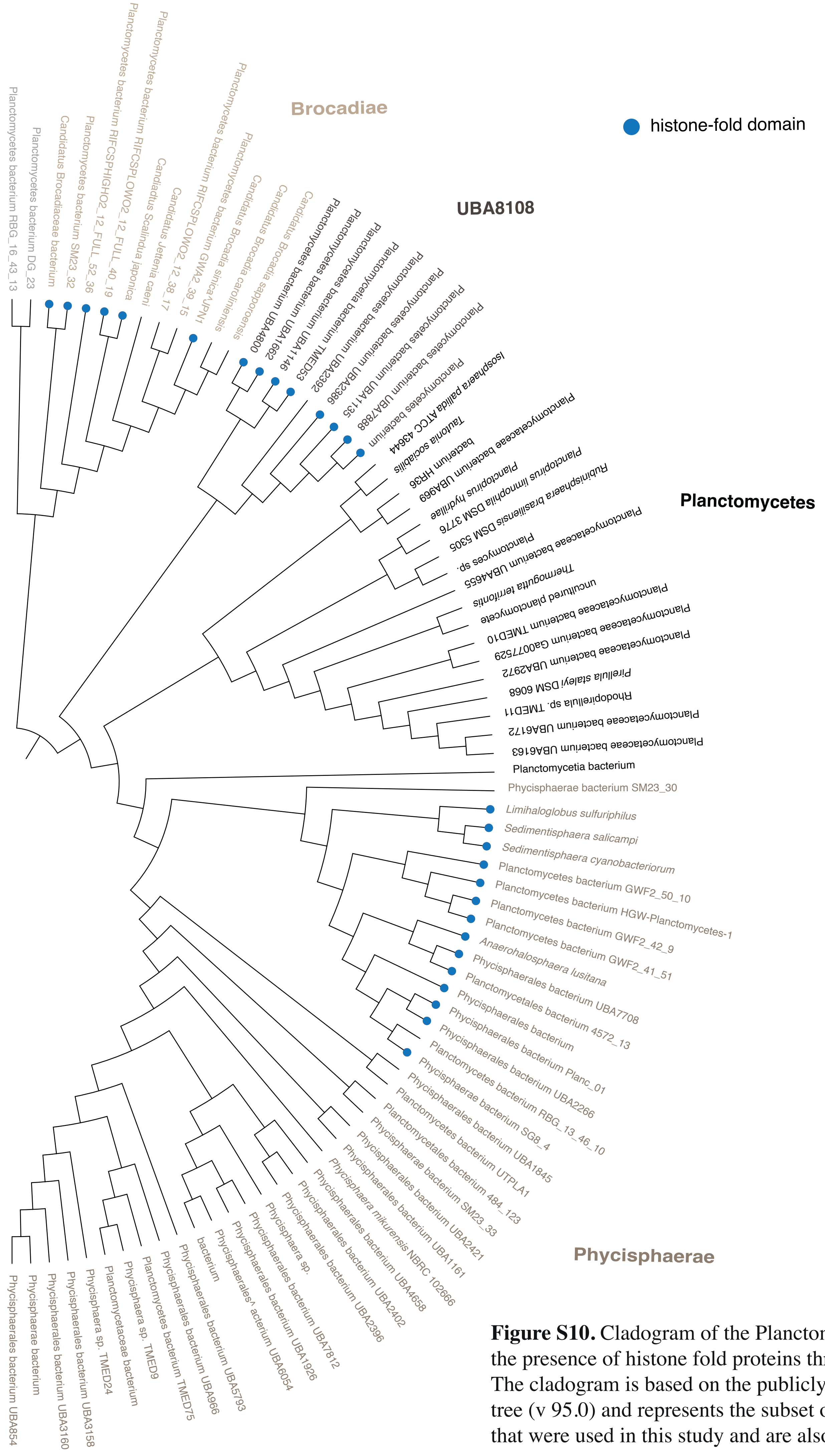

Figure S10. Cladogram of the Planctomycetota, indicating the presence of histone fold proteins throughout the clade. The cladogram is based on the publicly available GTDB tree (v 95.0) and represents the subset of genome assemblies that were used in this study and are also present in GTDB.

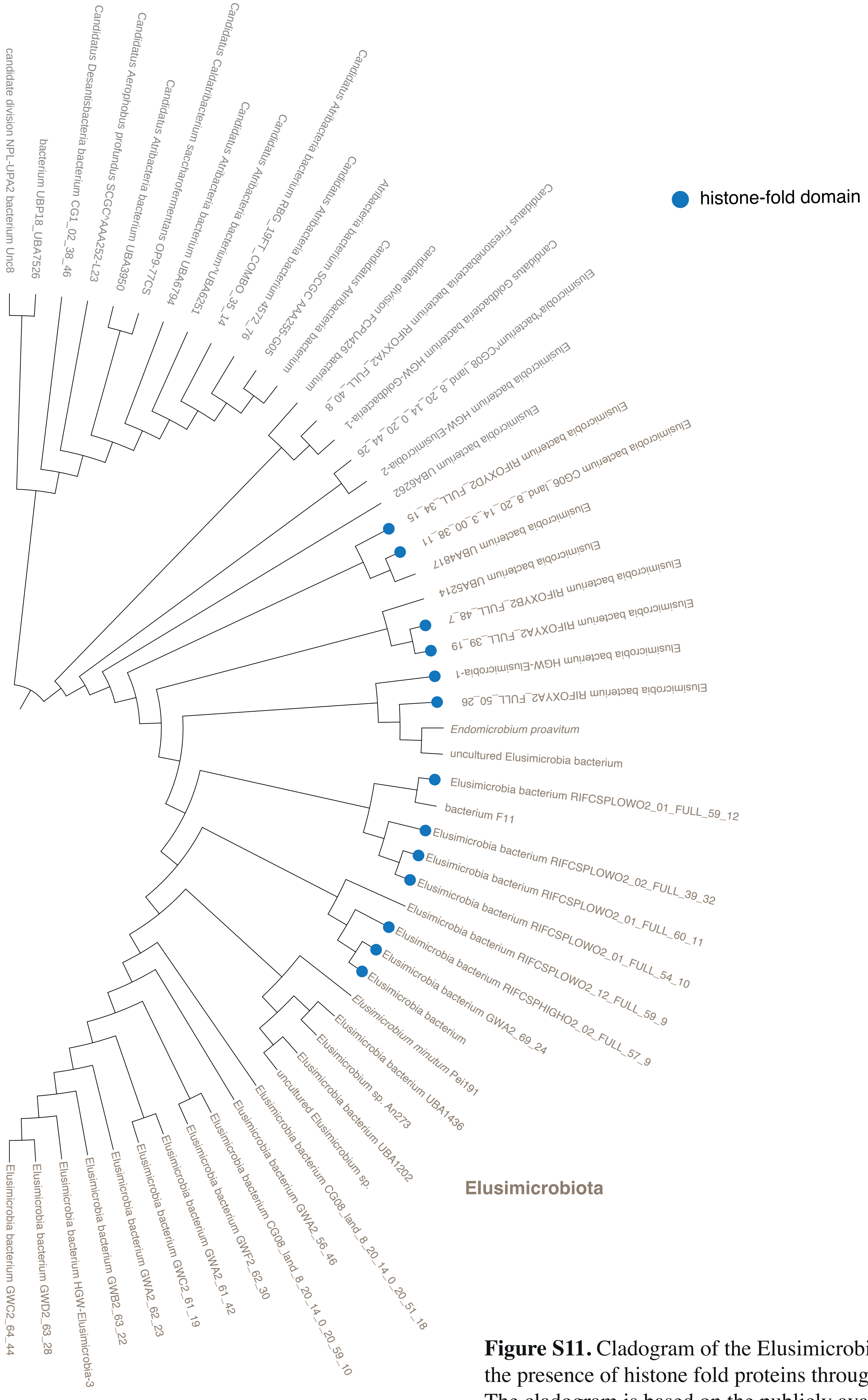

Figure S11. Cladogram of the Elusimicrobia, indicating the presence of histone fold proteins throughout the clade. The cladogram is based on the publicly available GTDB tree (v 95.0) and represents the subset of genome assemblies that were used in this study and are also present in GTDB.

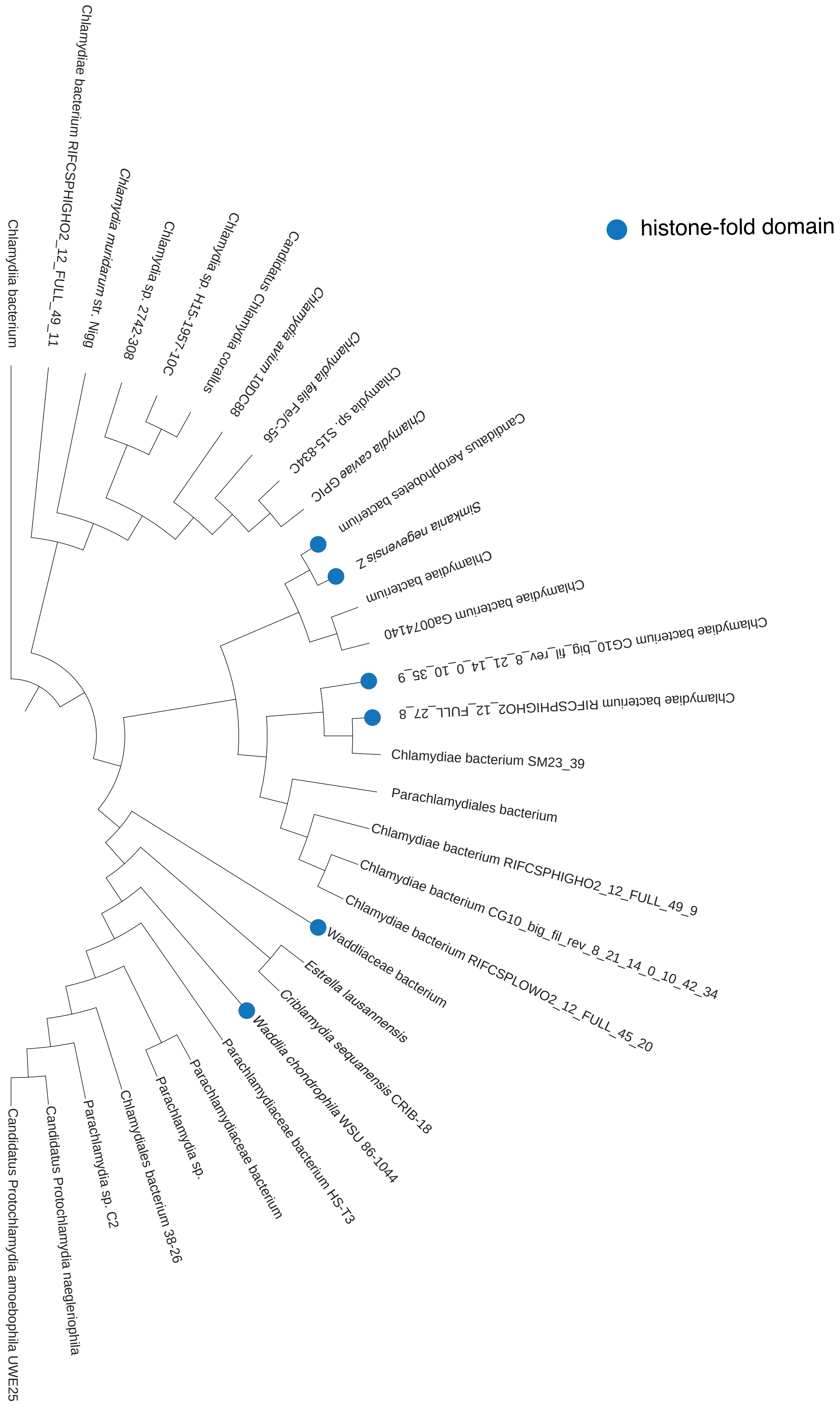

Figure S12. Cladogram of the Chlamydia, indicating the presence of histone fold proteins throughout the clade. The cladogram is based on the publicly available GTDB tree (v 95.0) and represents the subset of genome assemblies that were used in this study and are also present in GTDB.

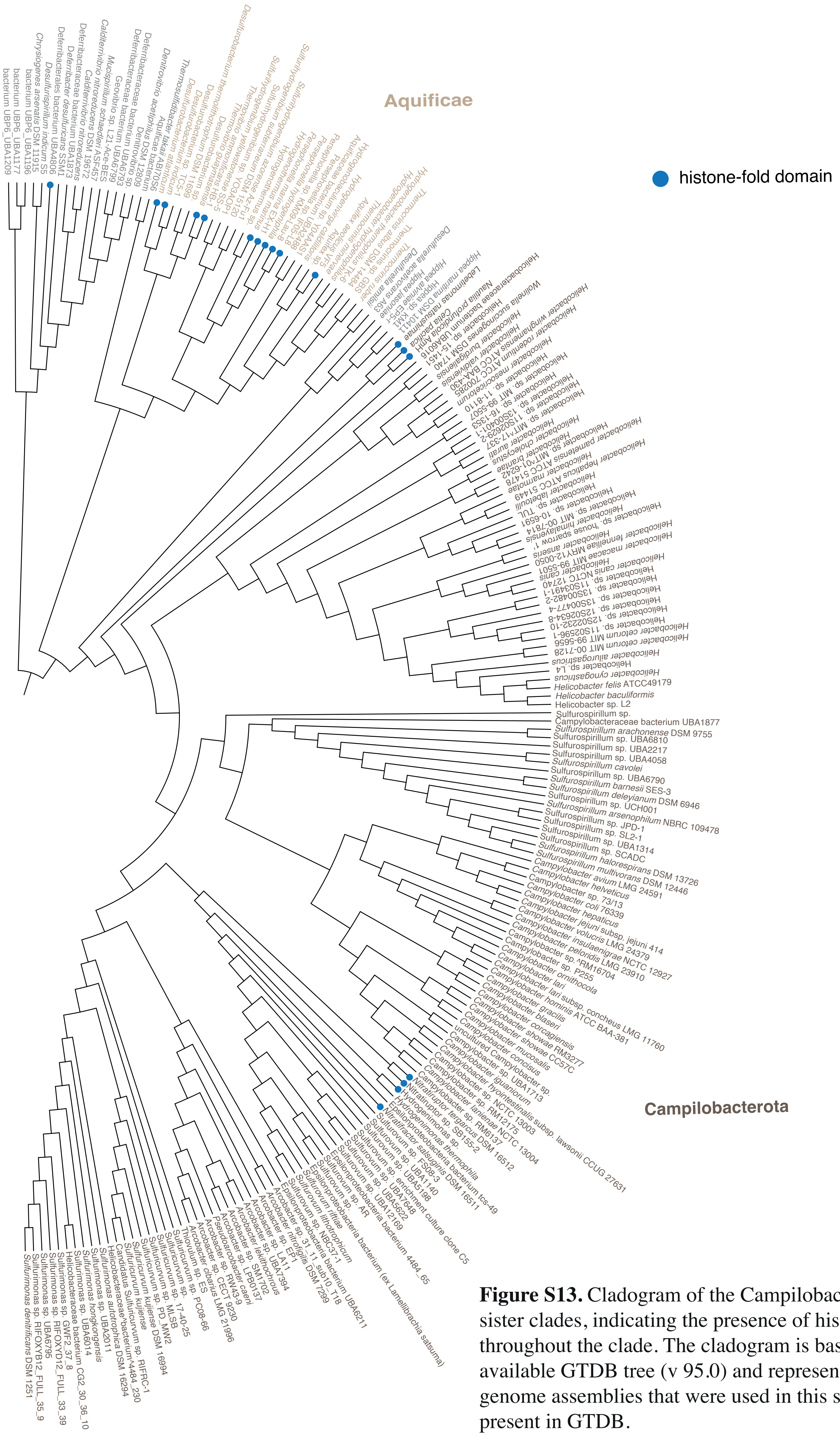

Figure S13. Cladogram of the Campilobacterota, Aquificae & sister clades, indicating the presence of histone fold proteins throughout the clade. The cladogram is based on the publicly available GTDB tree (v 95.0) and represents the subset of genome assemblies that were used in this study and are also present in GTDB.

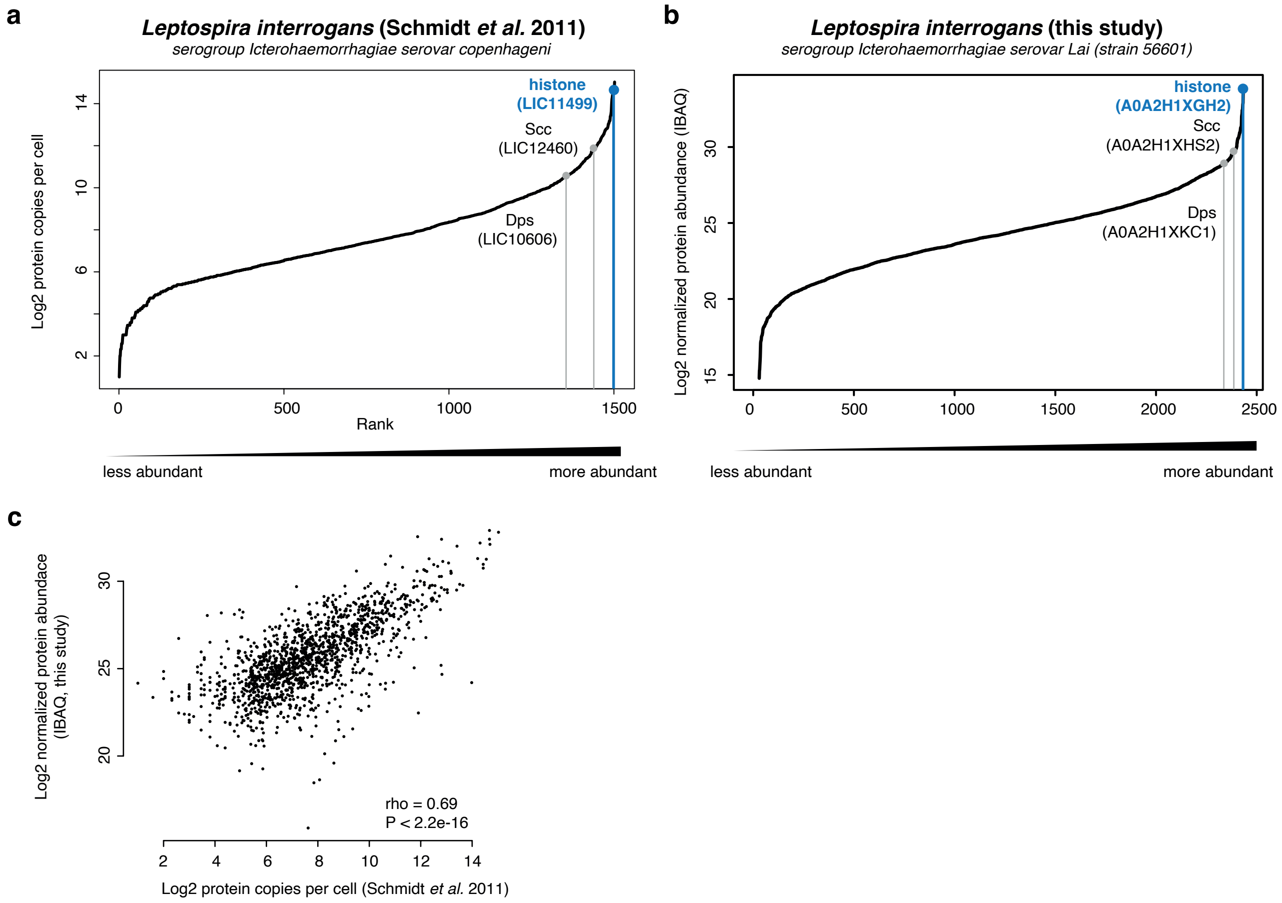

Figure S14. Ranked abundance of *L. interrogans* proteins from **(a)** Schmidt *et al*. (2011) and **(b)** this study, highlighting the abundance of the *L. interrogans* histone in the context of other known NAPs in this species. All quantified proteins are plotted. (**c**) Comparison between absolute protein abundance values reported in Schmidt *et al*. (2011, ref. 38) and normalized abundance values (IBAQ) reported in this study.

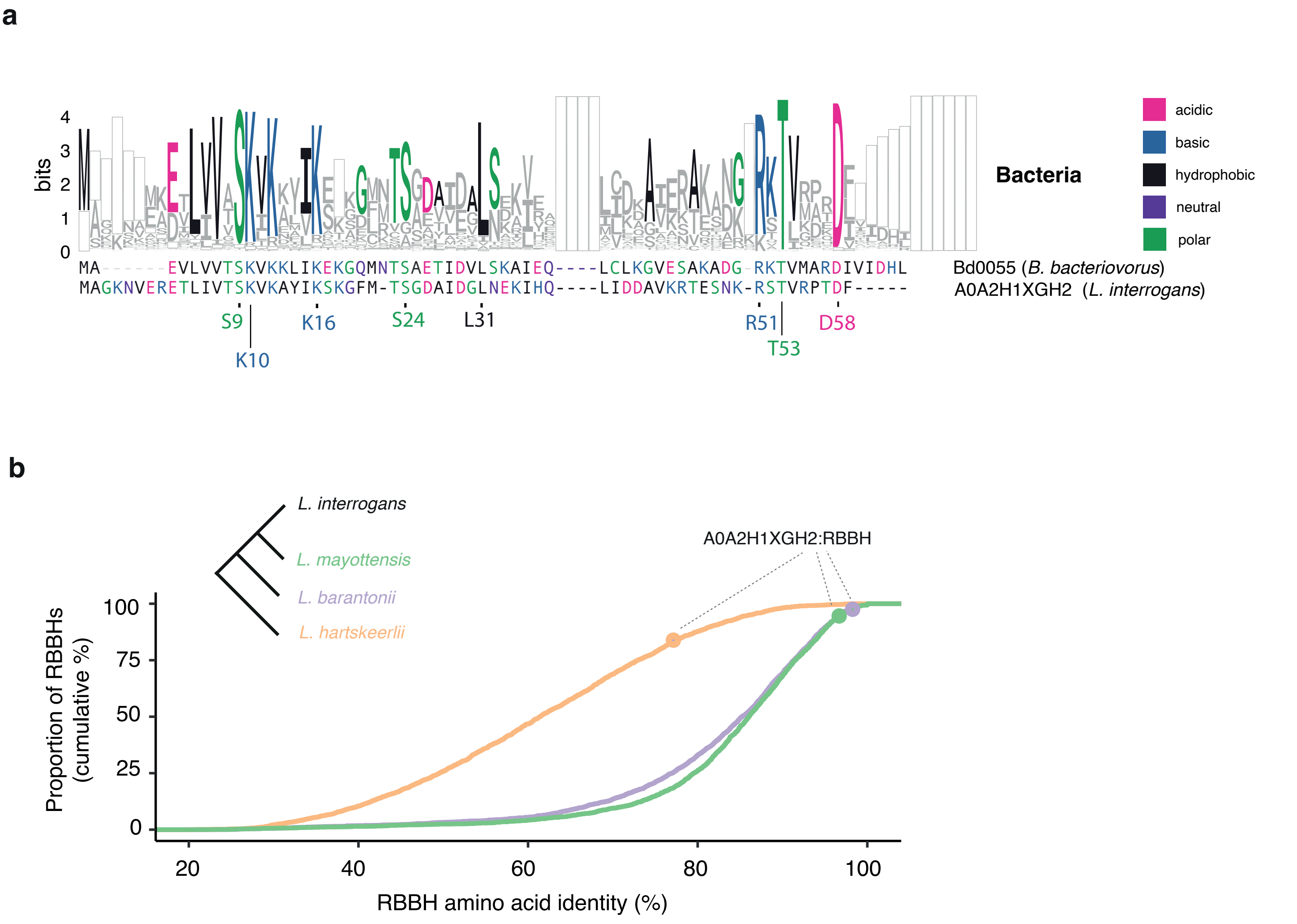

Figure S15. (a) Alignment of Bd0055 (*B. bacteriovorus*) and A0A2H1XGH2 (*L. interrogans*) in the context of overall amino acid usage across bacterial singlets (see Fig. 1c). **(b)** Conservation of the *L. interrogans* singlet histone (A0A2H1XGH2) at the amino acid level in the context of other proteins encoded by *L. interrogans*. Homologs of proteins in *L. interrogans* were identified in three other Leptospira genomes of different divergence levels (*L. mayottensis*, *L. barantonii*, *L. hartskeerlii*) using a reciprocal best blast hit approach (see Methods). For each pairwise comparison, proteins were aligned and then ranked by amino acid identity. A0A2H1XGH2 ranks amongst the most highly conserved proteins at different levels of proteome divergence.

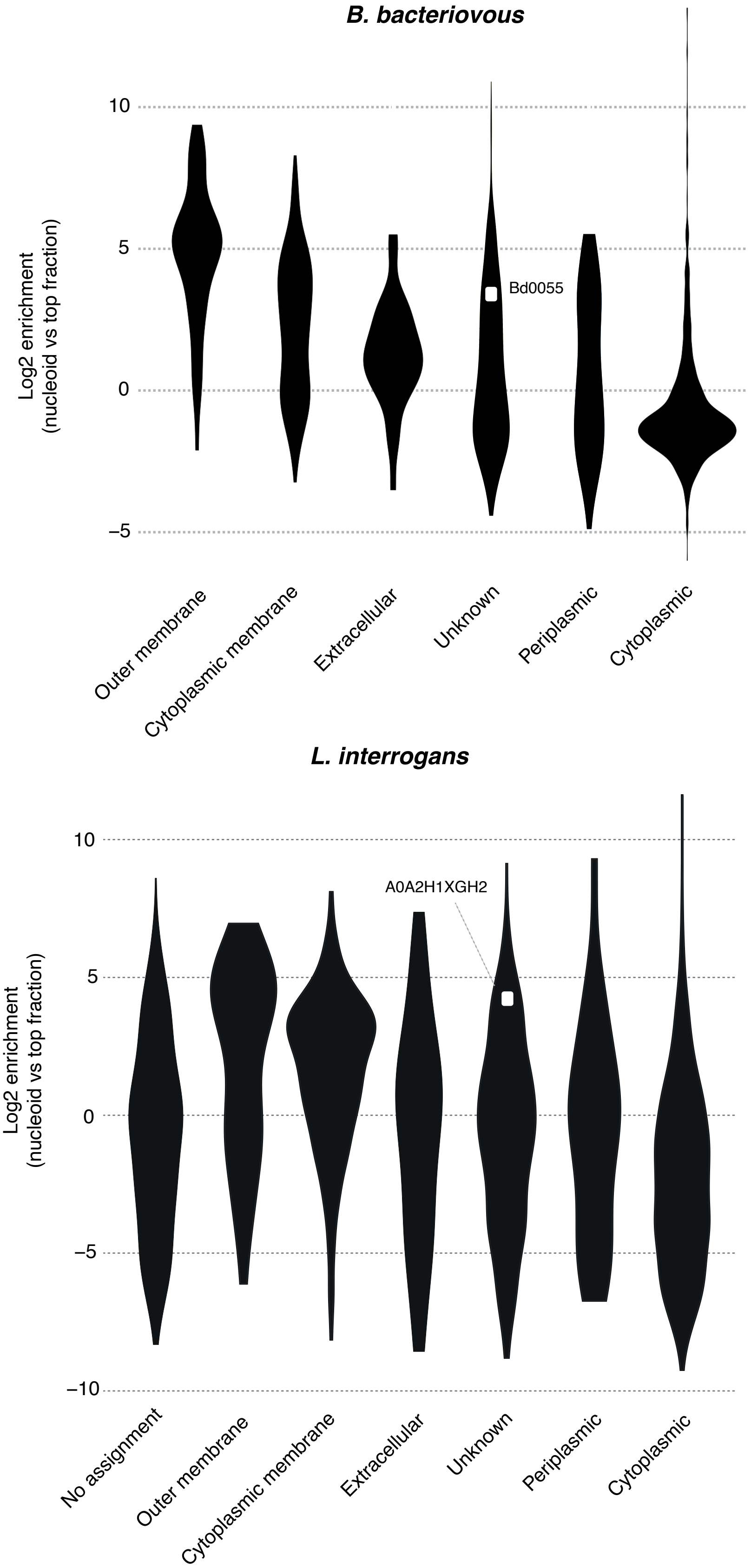

Figure S16. Nucleoid enrichment of proteins in *B. bacteriovorus* and *L. interrogans* as a function of their cellular localization as predicted by PSORTdb (https://db.psort.org/).

Table S2. X-ray diffraction data collection and refinement statistics (MR).

	Bd0055 apo	Bd0055 bound to 35 bp dsDNA
**Data collection**		
Space group	P 2 21 21	I 21 21 21
Cell dimensions		
a, *b*, *c* (Å)	26.7722 34.4118 56.484	33.081 103.143 111.145
α, β, γ (°)	90 90 90	90 90 90
Resolution (Å)	29.39 - 1.8 (1.865 - 1.8)	27.79 - 2.0 (2.072 - 2.0)
R_{merge}	0.05163 (0.1029)	0.06045 (0.5857)
$I / \sigma I$	26.79 (10.42)	16.49 (3.86)
Completeness (%)	99.42 (95.25)	98.37 (97.89)
Redundancy	8.4 (5.2)	7.1 (7.4)
**Refinement**		
No. reflections	5,156 (461)	13,136 (1,302)
R_{work}	0.1889 (0.2095)	0.2218 (0.2320)
R_{free}	0.2331 (0.2670)*	0.2536 (0.2508)
No. atoms		
Macromolecules	476	1051
Ligand	0	0
Solvent	43	52
Protein residues	63	126
B-factors		
Macromolecules	11.88	46.07
Solvent	20.65	42.45
R.m.s. deviations		
Bond lengths (Å)	0.007	0.008
Bond angles (°)	0.82	0.92

Values in parentheses are for highest-resolution shell. One crystal was used for each structure. *Two N-terminal residues (Ala 2 and Glu 3) are modeled in for completeness despite being rather disordered. The R_{work} and R_{free} values when those two residues are removed are 0.1932 and 0.2259, respectively.

Supplementary References

66. Karunker, I. *et al.* A global transcriptional switch between the attack and growth forms of *Bdellovibrio bacteriovorus*. *PLoS One* 8:e61850, (2013).

67. Yin, R. *et al.* Benchmarking AlphaFold for protein complex modeling reveals accuracy determinants. *Prot. Sci.* 31, e4379, (2022).
