# Supplementary material for: Histones with an unconventional DNA-binding mode in vitro are major chromatin constituents in the bacterium Bdellovibrio bacteriovorus
Source: Nat Microbiol. 2023 Oct 9;8(11):2006–19. doi: 10.1038/s41564-023-01492-x (PMC10627809; doi:10.1038/s41564-023-01492-x)
Supplement: Supplementary file 2 — Reporting Summary [file 41564_2023_1492_MOESM2_ESM.pdf]

nature portfolio

Corresponding author(s): Antoine Hocher, Karolin Luger, Tobias Warnecke

Last updated by author(s): Aug 18, 2023

Reporting Summary

Nature Portfolio wishes to improve the reproducibility of the work that we publish. This form provides structure for consistency and transparency in reporting. For further information on Nature Portfolio policies, see our Editorial Policies and the Editorial Policy Checklist.

Statistics

For all statistical analyses, confirm that the following items are present in the figure legend, table legend, main text, or Methods section.

n/a	Confirmed	
☐	☒	The exact sample size (*n*) for each experimental group/condition, given as a discrete number and unit of measurement
☐	☒	A statement on whether measurements were taken from distinct samples or whether the same sample was measured repeatedly
☐	☒	The statistical test(s) used AND whether they are one- or two-sided<br>*Only common tests should be described solely by name; describe more complex techniques in the Methods section.*
☐	☒	A description of all covariates tested
☒	☐	A description of any assumptions or corrections, such as tests of normality and adjustment for multiple comparisons
☐	☒	A full description of the statistical parameters including central tendency (e.g. means) or other basic estimates (e.g. regression coefficient) AND variation (e.g. standard deviation) or associated estimates of uncertainty (e.g. confidence intervals)
☐	☒	For null hypothesis testing, the test statistic (e.g. *F*, *t*, *r*) with confidence intervals, effect sizes, degrees of freedom and *P* value noted<br>*Give P values as exact values whenever suitable.*
☒	☐	For Bayesian analysis, information on the choice of priors and Markov chain Monte Carlo settings
☒	☐	For hierarchical and complex designs, identification of the appropriate level for tests and full reporting of outcomes
☐	☒	Estimates of effect sizes (e.g. Cohen's *d*, Pearson's *r*), indicating how they were calculated

Our web collection on statistics for biologists contains articles on many of the points above.

Software and code

Policy information about availability of computer code

Data collection	Crystallography: XDS, HKL3000; Rigaku XtaLAB MM003<br>Mass spectrometry : Ultimate 3000 RSLC nano liquid chromatography system (Thermo Scientific), LTQ Orbitrap Velos & Orbitrap Q-Exactive (Thermo Scientific)<br>Sequencing : MiSeq (Illumina); NextSeq 2000 (Illumina)
Data analysis	HMMER (v 3.1b2); Prodigal (v 2.6.3); Jpred4; iTol (v 6.7); R (libraries: metablastr (v 0.3), DEP (v 1.8.0)); MaxQuant (v 1.6.10.43); pFind (v 3); Bowtie2 (v 2.4.4); BWA (v 0.7.17); HTSeq (v 0.6.1); PHENIX (v 1.20); DIALS (v 3.14); Coot (v 0.9.8.5); ChimeraX (v 1.6); Chimera (v 1.17.3); Alphafold (v 2.3.2)

For manuscripts utilizing custom algorithms or software that are central to the research but not yet described in published literature, software must be made available to editors and reviewers. We strongly encourage code deposition in a community repository (e.g. GitHub). See the Nature Portfolio guidelines for submitting code & software for further information.

Data

Policy information about availability of data

All manuscripts must include a data availability statement. This statement should provide the following information, where applicable:

- Accession codes, unique identifiers, or web links for publicly available datasets
- A description of any restrictions on data availability
- For clinical datasets or third party data, please ensure that the statement adheres to our policy

All datasets are publicly available. Proteomics data have been deposited to PRIDE (PXD039405) and RNA-seq data to GEO under accession GSE220534. All genomes used were publicly available with no usage restriction (Table 53). Structure data have been deposited at the Protein Data Bank (PDB 8FVX and 8FW7).

Research involving human participants, their data, or biological material

Policy information about studies with human participants or human data. See also policy information about sex, gender (identity/presentation), and sexual orientation and race, ethnicity and racism.

Reporting on sex and gender	NA
Reporting on race, ethnicity, or other socially relevant groupings	NA
Population characteristics	NA
Recruitment	NA
Ethics oversight	NA

Note that full information on the approval of the study protocol must also be provided in the manuscript.

Field-specific reporting

Please select the one below that is the best fit for your research. If you are not sure, read the appropriate sections before making your selection.

☒ Life sciences ☐ Behavioural & social sciences ☐ Ecological, evolutionary & environmental sciences

For a reference copy of the document with all sections, see nature.com/documents/nr-reporting-summary-flat.pdf

Life sciences study design

All studies must disclose on these points even when the disclosure is negative.

Sample size	Sample size for biochemical/simulation experiments was n=3. Variance across experiments was low so explicit power calculations were not considered necessary.
Data exclusions	For FP and FRET experiments data was excluded at high protein concentration, where salt from the lM NaCl storage buffer had large effects (above 12.SuM protein) and below ~1nM protein, as all curves had flattened out by this point.
Replication	Experiments were replicated on different days (N=3 in every case). All replication attempts were successful.
Randomization	Random allocation with regard to covariates is not applicable to the experiments carried out.
Blinding	Blinding was not relevant for any of the experiments carrier out in this study.

Reporting for specific materials, systems and methods

We require information from authors about some types of materials, experimental systems and methods used in many studies. Here, indicate whether each material, system or method listed is relevant to your study. If you are not sure if a list item applies to your research, read the appropriate section before selecting a response.

Materials & experimental systems

n/a	Involved in the study
☒	☐ Antibodies
☒	☐ Eukaryotic cell lines
☒	☐ Palaeontology and archaeology
☒	☐ Animals and other organisms
☒	☐ Clinical data
☒	☐ Dual use research of concern
☒	☐ Plants

Methods

n/a	Involved in the study
☒	☐ ChIP-seq
☒	☐ Flow cytometry
☒	☐ MRI-based neuroimaging
